# Predictors of Mortality for Nursing Home-Acquired Pneumonia: A Systematic Review

**DOI:** 10.1155/2015/285983

**Published:** 2015-03-02

**Authors:** Naveen Dhawan, Naushira Pandya, Michael Khalili, Manuel Bautista, Anurag Duggal, Jaya Bahl, Vineet Gupta

**Affiliations:** ^1^Nova Southeastern University Health Sciences Division, Fort Lauderdale, FL 33314, USA; ^2^The Commonwealth Medical College, Scranton, PA 18509, USA; ^3^Division of Pulmonary Medicine, Northeast Ohio Medical University College of Medicine, Rootstown, OH 44272, USA; ^4^Division of Infectious Diseases, Medina Hospital (Cleveland Clinic), Medina, OH 44256, USA; ^5^Division of Infectious Diseases, Northeast Ohio Medical University College of Medicine, Rootstown, OH 44272, USA; ^6^Florida International University (FIU), North Miami Beach, FL 33181, USA; ^7^Department of Medicine, University of California San Diego (UCSD), 200 West Arbor Drive, San Diego, CA 92103, USA

## Abstract

*Background*. Current risk stratification tools, primarily used for CAP, are suboptimal in predicting nursing home acquired pneumonia (NHAP) outcome and mortality. We conducted a systematic review to evaluate current evidence on the usefulness of proposed predictors of NHAP mortality. *Methods*. PubMed (MEDLINE), EMBASE, and CINAHL databases were searched for articles published in English between January 1978 and January 2014. The literature search elicited a total of 666 references; 580 were excluded and 20 articles met the inclusion criteria for the final analysis. *Results*. More studies supported the Pneumonia Severity Index (PSI) as a superior predictor of NHAP severity. Fewer studies suggested CURB-65 and SOAR (especially for the need of ICU care) as useful predictors for NHAP mortality. There is weak evidence for biomarkers like C-reactive protein and copeptin as prognostic tools. *Conclusion*. The evidence supports the use of PSI as the best available indicator while CURB-65 may be an alternative prognostic indicator for NHAP mortality. Overall, due to the paucity of information, biomarkers may not be as effective in this role. Larger prospective studies are needed to establish the most effective predictor(s) or combination scheme to help clinicians in decision-making related to NHAP mortality.

## 1. Introduction

The rate of growth in the elderly population around the world has led to the estimate that 40% of adults around the world will reside in a long-term care (LTC) facility for some time before death, over the next 30 years [1]. Nursing home-acquired pneumonia (NHAP) is currently the second most common type of infection among patients in LTC facilities in the USA [2, 3]. NHAP is also responsible for a majority of emergency department transfers [2]. Several patient characteristics predispose nursing home patients to pneumonia, including chronic diseases, impaired functional abilities, malnourishment, diminished cough reflex, lack of elastic tissue, and decreased immunoglobulin A [1, 4–6]. Additionally, attributes of the nursing home setting including the lack of immunizations, presence of multi-drug-resistant organisms, and widespread use of antibiotics also contribute to a greater risk of pneumonia [7]. Thus, pneumonia represents 13–48% of all infections in nursing home settings [8, 9].

Importantly, NHAP has the highest rates of morbidity and mortality among all the infections that occur in nursing homes, with rates of mortality reported to reach 55% [8, 9]. In some countries such as South Korea, NHAP is the leading cause of mortality in long-term residents [10]. The incidence of pneumonia is estimated to be 10 times greater in nursing homes compared to individuals living in the community [11]. The hospitalization rates of NHAP, however, are estimated to be almost 30 times that of CAP [12]. The significance of NHAP mortality is illustrated by the fact that NHAP mortality rates are higher than those related to community-acquired pneumonia (CAP), ranging from 5 to 40% according to some accounts [2, 12, 13]. While traditionally the excess mortality of NHAP was thought to be due to multi-drug-resistant (MDR) organisms, current research has pointed to the presence of comorbidities in the nursing home setting as a major reason for the higher mortality than CAP [14].

The nursing home setting poses several diagnostic challenges for identifying NHAP. While a causative agent is identified less than 50% of the time in elderly pneumonia patients, in nursing homes the percentage is even less; sputum production from patients for samples is challenging due to delirium, dementia, impaired cough reflex, and dehydration [15]. Other obstacles in obtaining essential respiratory samples include the fact that many patients are treated with empirical antibiotics or steroids prior to specimen collection [2, 13, 16]. The interpretation of signs and symptoms is often confounded by several factors. Chest radiography is often challenging to conduct in the elderly due to numerous factors: the relative suboptimal quality of portable radiographs used in nursing homes and the difficulty for frail patients to cooperate and stay upright for X-rays [17]. Additionally, elderly patients present with atypical presentations and with less symptoms than younger patients, complicating the recognition of NHAP [2, 17–19]. Nursing home patients typically are afflicted with several comorbidities, including depression, declining functional status, chronic heart failure, impaired cognition, and respiratory ailments; thus clinical symptomology and laboratory results often lack sensitivity and specificity for NHAP [20]. Thus, there is often a delay in the diagnosis of pneumonia, thereby contributing to increased mortality in elderly patients [21]. This warrants the need for validating optimal clinical biomarkers and clinical tools to diagnose and assess the severity of NHAP.

Yet, clinicians lack a definitive prognostic tool or risk stratification scheme that distinguishes which nursing home residents may be more prone to mortality due to NHAP [22]. In recent years, a growing area of interest has been identifying the most optimal biomarkers in the management and prognosis of NHAP. The fact that guidelines among infectious disease societies differ with antimicrobial approach (as some focus on empirical treatment covering drug-resistant pathogens) underscores the importance of a severity assessment scheme that can influence medication decisions [16]. Recognition of disease severity and identification of patient subtypes that may be at a greater risk of mortality can influence decisions on antibiotic administration.

An important issue that has been raised is whether it is necessary to hospitalize all NHAP patients. There is a clear need for a clinical tool that can predict mortality and functional deterioration due to hospitalization in NHAP patients. Such a tool would enhance patient outcomes and enable better allocation of healthcare resources. Additionally, a predictive tool may influence decisions for ICU admission for NHAP patients presenting to the emergency department setting [23]. This systematic review sought to explore the current literature on NHAP risk stratification and specific predictors for disease severity that may serve as a clinical judgment tool for clinicians and inform decisions of prioritizing care, hospitalization, ICU admissions, and administering prophylactic antibiotics.

## 2. Methods

The authors investigated NHAP with the goal of identifying predictors of mortality from this disease. The study methodology conformed to the Preferred Reporting Items for Systematic Reviews and Meta-Analyses (PRISMA) Statement for systematic reviews [24].

### 2.1. Eligibility Criteria

Studies were selected based on preset inclusion and exclusion criteria. Inclusion criteria are comprised of studies on NHAP with patients 65 years of age or older, assessing risk factors, and predictors of prognosis. Many studies have included younger individuals who have a different prognosis [14]. It has thus been argued that the attributes of NHAP can be best determined by comparing patients who are at 65 years of age or older [14].

### 2.2. Information Sources and Search

A literature search utilized several databases: PubMed, EMBASE, and CINAHL. Studies were selected based on preset inclusion and exclusion criteria. Systematic search was restricted to studies including humans and published in English, from January 1978 to January 2014. An experienced librarian in electronic search methods performed the literature search. The search strategy and keywords employed in this study are summarized in Table 1.

### 2.3. Study Selection and Data Collection Process

All abstracts were read and articles of potential interest were reviewed in detail (full text) by authors Michael Khalili and Naveen Dhawan to decide on inclusion or exclusion from this systematic review. In cases of disagreement, both authors reviewed and discussed the study and a final decision was made through consensus.

### 2.4. Data Extraction

Michael Khalili extracted information regarding NHAP from all included studies using a predetermined template comprising of type of study, number of patients, risk factors for NHAP, and predictors of NHAP severity. Given the heterogeneity of the characteristics studied in the included papers, a meta-analysis was not performed.

## 3. Results

### 3.1. Study Selection

**Figure 1:**
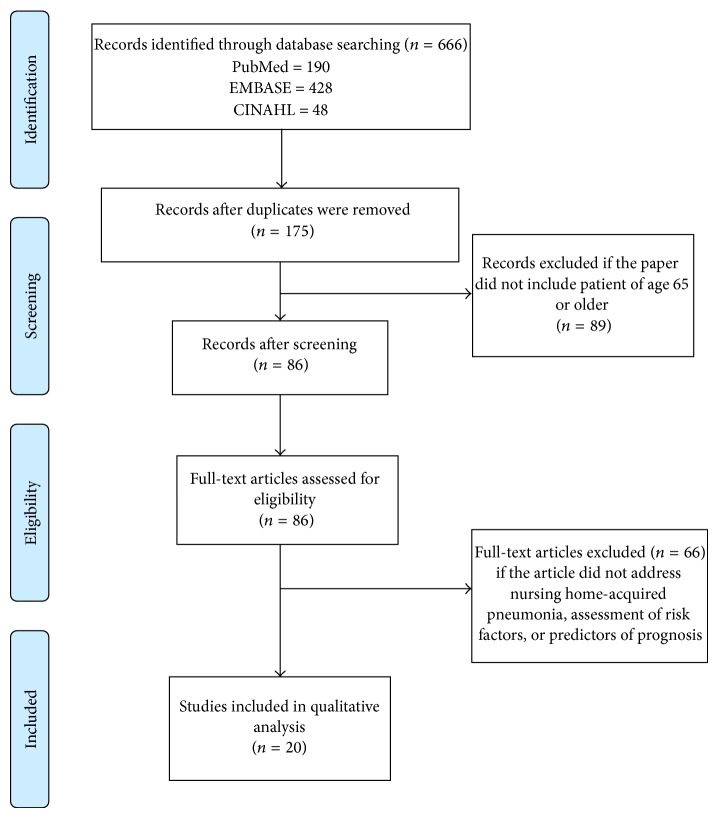
Study flow of the selection process of all papers used in the final analysis.

The literature search elicited a total of 666 references, from which 491 were duplicates and another 89 were excluded (Figure 1). A total of 86 articles were reviewed in full text; 20 articles met the inclusion criteria for the current systematic review that are presented in Table 2.

### 3.2. Characteristics of the Studies

Results of the data extraction of selected papers are presented in Table 2.

## 4. Discussion

### 4.1. Demographics

NHAP typically affects the elderly patient population that has multiple comorbidities [25]. Several studies that have investigated NHAP in hospitalized patients show mean ages to be 74–82 years, while the 30-day mortality is reported to range from 16.8% to 24.7% [16, 25–31]. A study in Germany by Klapdor et al. [25] utilizing prospective multicenter data examined 618 patients and compared an older subset (65 years or older) to those less than 65 years. In this study, 16% (100 patients) were aged 65 or older and the younger patients below 65 years of age had a mean age of 54 years [25]. While multi-drug-resistant (MDR) pathogens are a therapeutic challenge in NHAP overall incidence was low in the both groups. The younger subject group (less than 65 years of age) showed two times lower mortality than those 65 or over [25]. Importantly, Klapdor et al. showed that patient age was a significant determinant in presentation and prognosis of NHAP patients. In older patients, typical symptoms of pneumonia such as sputum production, cough, and fever are often absent [25]. They also found that the younger patients mostly presented with fever. Importantly, roughly two-thirds of those with NHAP display temperatures over 100.4 degrees Fahrenheit; nursing home patients have lower baseline temperatures and lower peak temperatures resulting from infection [13]. The diagnosis of pneumonia is often complicated due to delayed recognition stemming from preexisting and chronic conditions and comorbidities (i.e., stroke and dementia); thus, there is a higher rate of mortality in elderly patients [13, 21, 32]. Further, it has been shown that the prognosis for NHAP is worse for men than women [33].

### 4.2. Infective Etiology

The microbial infective etiology of NHAP remains controversial [2]. A particular challenge in determining microbial etiology lies in obtaining adequate sputum samples, since only as estimated 50–70% of nursing home patients are unable to produce decent sputum samples [2]. While antigen testing of respiratory secretions is useful in diagnosing viruses such as RSV and influenza, urinary antigen testing for* Legionella pneumophila* serotype 1 and* Streptococcus pneumonia* remains challenging due to limited knowledge regarding the susceptibility to antibiotics [34].

The infective agents in NHAP also vary throughout the world. For instance, in a recent prospective cohort study of 488 nursing home resident patients hospitalized for pneumonia, over half (55.9%) of NHAP cases were due to a viral infection [35]. Yet, in a recent prospective study of 217 nursing home residents, 56.3% of NHAP cases were due to Enterobacteriaceae [6]. In the USA, NHAP most commonly stems from bacteria yet the specific cause is often unknown [3]. While* Streptococcus pneumoniae* has been identified as the most common infective agent, in more severe NHAP resulting in hospitalization* Staphylococcus aureus* and the enteric Gram-negative agents appear more commonly than* Streptococcus pneumonia* [3, 36]. In a recent study,* Staphylococcus aureus* was reported to account for the highest mortality [14]. According to one study,* Staphylococcus *spp. were the most common agent in the USA comprising 52% of cases, while* Streptococcus pneumoniae* was the most prominent in Europe and Latin America (it was found in 46% of cases in Europe and 25% in Latin America) [37]. The large presence of multi-drug-resistant agents in NHAP patients in the USA has been documented [1, 2, 38].

**Figure 2:**
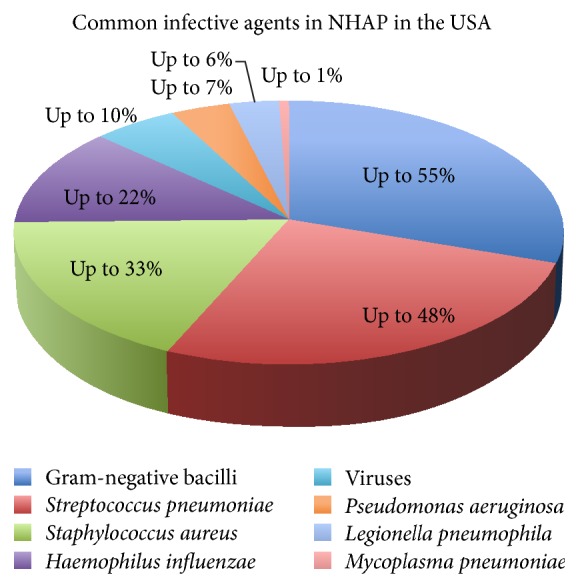
The most frequent infective etiology of NHAP (estimates) [3].

Typical bacterial agents in NHAP in the USA include* Staphylococcus aureus*,* Pseudomonas*,* Klebsiella*,* Proteus mirabilis*, and* E coli*. Typical community-acquired organisms that cause pneumonia include* Streptococcus pneumoniae*,* Haemophilus influenzae*,* Mycoplasma*,* Legionella*, and* Chlamydia*. The antibiotics used in CAP include azithromycin, macrolides, fluoroquinolones (i.e., levofloxacin), and a combination of a beta-lactam and a cephalosporin. Common causative organisms of NHAP in the USA are shown in Figure 2.

The infective etiology differs, however, in countries such as Poland. In a recent prospective study of 217 patients age 65 or above, 56.3% of microorganisms are comprised of Enterobacteriaceae, 25%* Pseudomonas aeruginosa*, and* Staphylococcus aureus* 12.5%, and* Candida albicans* is comprised of 6.3% [6]. Thus, NHAP and CAP share many features in their microbial infective etiology including the emergence of drug-resistant bacteria. Ma et al. [35] suggested that NHAP should not be treated as healthcare-associated pneumonia instead of community-acquired pneumonia (CAP). The researchers found that, in both NHAP and CAP, multi-drug-resistant bacteria were not common [35]. Importantly, NHAP can also be caused by a viral etiology [3]. Respiratory syncytial virus (RSV) and influenza remain the most common sources of respiratory disease and fatality in nursing homes [39, 40].

### 4.3. Treatment Guidelines for NHAP

There is no clear consensus in the management of NHAP. The IDSA and Canadian Infectious Disease Society have put forth similar guidelines for antibiotic use in CAP that discuss pneumonia treatment in long-term healthcare facilities; both societies recommend a beta-lactam in combination with a macrolide or an antipneumococcal fluoroquinolone antibiotic by itself [41, 42]. British Thoracic Society (BTS) 2009 pneumonia guidelines mention increased risk of aspiration pneumonia in nursing home patients [43]. Yet, these guidelines do not include specific guidance for NHAP treatment in the nursing home setting [2].

A major discrepancy exists between the established NHAP guidelines for treatment by the 2003 Infectious Disease Society of America (IDSA) and the 2005 American Thoracic Society/IDSA recommendations regarding etiological agents and treatment focus; the 2005 American Thoracic Society/IDSA suggests empiric treatment as it recognizes drug-resistant organisms (*Pseudomonas aeruginosa* and MRSA) to be main causative agents [41, 44].

NHAP is generally treated with broad-spectrum antibiotics, with the consideration of these drug-resistant bacteria [3]. The treatment of NHAP is generally distinguished by whether patients are treated in the nursing home or are hospitalized with NHAP. In nursing homes, treatment entails either an antipneumococcal fluoroquinolone (i.e., levofloxacin or moxifloxacin) by itself or a combination of either a high level dosage beta-lactamase or beta-lactam inhibitor (i.e., Augmentin) with azithromycin, or a combination of a second or third generation cephalosporin with azithromycin [3]. Patients treated in the hospital setting with NHAP are treated with broad-spectrum agents that cover several Gram-positive and Gram-negative bacteria (i.e., MRSA) [3]. Accurate dosing is important for averting potential side effects and drug interactions with other medications must be considered [3].

Importantly, doxycycline has been proposed as a potential alternate drug for treating nonhospitalized NHAP patients [2]. Doxycycline has advantages including the ability to counter atypical pathogens (i.e.,* Legionella *and* Chlamydophila pneumoniae*) and penicillin-resistant* Streptococcal pneumoniae*, good oral absorption, and lower cost than fluoroquinolones [2]. However, its drug interactions and restricted Gram-negative activity prevent it from wide use in nursing home patients, and no study has supported its use as a sole therapeutic agent in treating NHAP [2].

### 4.4. Clinical Predictors

Compared to CAP, applying a definitive set of clinical predictors for NHAP risk of death is complicated by the multiple comorbidities that characterize nursing home patients [12]. Interestingly, it has been shown that a combination of fever, cough, shortness of breath, and elevated WBCs is not as accurate in the diagnosis of NHAP as it is in CAP [20]. Yet, several clinical attributes have been posed for NHAP prognosis. It has been shown that suboptimal nutritional status and comorbidities in the elderly are independently correlated with poor outcomes in institutionalized elderly who suffer from aspiration pneumonia [45]. Elderly age has been shown to correlate with a poor pneumonia prognosis. In a retrospective study of 618 patients that compared two age groups, Klapdor et al. [25] found that short- and long-term mortality were twice as high in the elderly (age 65 or above) population compared to the younger group (under age 65) [25].

Patients with severe dementia are more likely to have a greater risk of mortality from pneumonia. In a prospective cohort study that assessed the severity of dementia and suboptimal prognoses in NHAP, van der Steen et al. [46] showed that dementia severity is an independent risk factor for mortality following pneumonia; the risk of mortality in the upper 25% of severe pneumonia patients had a three times higher risk of 1-week mortality than the bottom 25% of severity, while the risk was 2.5 times greater for 3-month mortality [46].

### 4.5. Prognostic Scoring Tools

Several prognostic scoring tools have been established for CAP, and while these have been proposed for use in NHAP, their use in NHAP is not clearly established [23]. The most widely used indices for predicting 30-day mortality are the CURB-65 and the Pneumonia Severity Index (PSI) [47, 48]. The CURB-65 score, developed by the British Thoracic Society, is used to predict the 30-day mortality from CAP and aids clinicians in decisions for outpatient versus inpatient treatment in patients over 65 years of age [49, 50]. The score takes into account five parameters: confusion, BUN levels above 19 mg/dL (or 7 mmol/L), respiratory rate equal to or over 30 per minute, a systolic blood pressure less than 90 mmHg or diastolic blood pressure equal to or less than 60 mmHg, and patients age greater than or equal to 65 years. The score ranges from 0 to 5, with increasing severity: one point is ascribed to each category; and a score equal to or greater than 2 is associated with a greater risk for mortality [51].

The Pneumonia Severity Index (PSI) considers up to 20 variables and has also been used in predicting CAP mortality [49]. In CAP, the PSI is known to have a better ability to predict low probability of mortality compared to CURB-65, which is better at predicting greater probability; however, CURB-65 does not take into account comorbidities [52, 53]. Lee et al. [10] demonstrated that the PSI had the best discriminatory power in the prediction of 30-day mortality, intensive vasopressor or respiratory support (IVRS), and severe pneumonia compared to the NHAP score, SOAR, and CURB-65. However, it is important to note that these results were based on the Korean patient population. Also, applying the PSI for nursing home patients presents a challenge; the PSI considers factors that require checks within the hospital setting (i.e., hematocrit, BUN, glucose levels in serum, sodium levels, arterial pH, pressure of oxygen, and X-ray findings) and may not be measured in many nursing homes that are not equipped with the necessary diagnostic and laboratory equipment [10]. It has also been noted that factors within the CURB-65 scoring such as confusion and a rise in blood urea are common occurrences in the elderly due to various acute illnesses and may not always be indicative of pending mortality [2].

In one prospective observational study of 767 NHAP patients in the emergency department of a teaching hospital in Hong Kong, Man et al. [23] found that PSI and CURB-65 were the most useful in determining patients suffering from less severe NHAP, as they serve to exclude severe cases. In a hospital-based study of 58 patients in Cyprus, Greece, Porfyridis et al. [20] found that procalcitonin and CURB-65 were the most accurate predictors of NHAP inpatient mortality.

Several prediction scales specific to the nursing home setting have been proposed in recent years. Naughton et al. [54] developed a 30-day mortality prediction model, the NHAP model score, based on a retrospective review of 378 NHAP episodes from 11 nursing homes. This model entails four main predictors: pulse greater than 125/min, respiration rate greater than 30/min, history of dementia, and altered mental status [54]. An increasing NHAP model score corresponds to greater mortality [10]. In a retrospective observational study of 208 nursing home patients with pneumonia by Lee et al. [10], the investigators provided a more rigorous study than the one by Naughton et al. [54] in evaluating the NHAP model. Lee et al. [10] confirmed the results of Naughton et al. [54] but showed that the NHAP model score fell behind PSI, CURB-65, and SOAR in terms of predictive value of 30-day mortality, IVRS, and severe pneumonia.

The Missouri Lower Respiratory Tract Infection Project was developed as a prediction tool for 30-day mortality stemming from infections in the lower respiratory tract; it acts as a factor in vitals, BMI, laboratory data, and daily activities [2]. Its utility lies in that, unlike other prediction tools, it is not dependent on X-ray assessment, yet one drawback is that it requires blood samples [2]. The tool has not been validated on a wide scale.

The SOAR scale (systolic blood pressure, oxygenation, age, and respiratory rate) has been developed to determine severe pneumonia in patients of advanced elderly age [55]. El-Solh et al. [50] studied 457 nursing home patients that were hospitalized at two university tertiary care centers and reported that SOAR had better predictive value in identifying patients in need of ICU admission than CURB-65 and CURB [50].

Another recent prediction tool, SMART-COP (systolic blood pressure, multilobar lung involvement, albumin, respiratory rate, tachycardia, confusion, O2, and arterial pH), has been used in CAP and proposed for use in NHAP [56]. España et al. [57] established a prediction rule based on 8 factors. Yet these prediction tools require further investigation.

Other prediction rules such as the modified American Thoracic Society rule (M-ATS) and the revised criteria (R-ATS) suggested by the Consensus Guidelines have been used in CAP but have not been widely studied in NHAP until recent years [34, 58, 59]. According to the study by Man et al. [23], the M-ATS and R-ATS rules show accuracy in the exclusion of severe pneumonia in patients less than or equal to 90 years that are functionally intact. Yet, these rules have not been validated on a large scale.

The Clinical Pulmonary Infection Score (CPIS) has been shown to have utility in diagnosing CAP and ventilator-acquired pneumonia (VAP) [60, 61]. This scale considers 6 factors (chest X-ray, secretions of trachea, WBC, temperature, microbial etiology, and PaO2/FiO2 ratio). In their study, Porfyridis et al. [20] confirmed that CPIS has reliability in diagnosing NHAP in its early stages. Yet, the role of CPIS as a severity predictor has not been established. A primary problem with these clinical tools is that they do not factor the functional status of the patient [62]. Interestingly, functional status is not an independent determinant of mortality in NHAP patients. Lower functional status in this subset of patients is associated with higher do-not-resuscitate code status that may explain higher mortality rates [63]. Additionally, these predictive tools do not take into account details of the host immune response that may be critical to a clinician's assessment and decision-making related to hospital admission [52].

### 4.6. Biomarkers

Biomarkers serve as an alternate, or even complimentary, form of prognostic prediction for NHAP. The rationale underlying the use of biomarkers is that since exorbitant amounts of cytokines have adverse effects on the body that lead to fatality, by measuring proinflammatory and anti-inflammatory cytokines as a degree of the inflammatory response of the host, we can assess the patients' mortality [52, 64–66]. Thus, their advantage over prognostic scoring tools lies in providing a quicker and more practical assessment of the host inflammatory response [52].

Yet, the selected studies showed scarce information overall supporting the use of biomarkers. Since many elderly patients present with fewer signs and symptoms and several cases are marked by atypical presentations, biomarkers often become more useful than prognostic scoring [18, 19, 67]. Thus, the advantage of serum biomarkers for NHAP patients includes the fact that may be more reliable since the presentation of NHAP is not specific; they are also not as complicated and difficult to apply as the scoring systems [17]. However, measuring serum levels of markers takes time and may not be feasible in resource-restricted health settings. Several biomarkers have been studied, including CRP, procalcitonin, proatrial natriuretic peptide, provasopressin, carboxy-terminal provasopressin (copeptin), and adrenomedullin [17]. Procalcitonin (PCT), the prehormone of calcitonin secreted by thyroid C cells, has been studied for its presence during inflammatory processes [67]. Porfyridis et al. [20] demonstrated that PCT had a better correlation in the prediction of in-hospital mortality for NHAP. Yet PCT remains problematic as a diagnostic marker and the study results of Kim et al. [67] confirm that PCT may serve as a poor marker in NHAP.

C-reactive protein (CRP), an acute phase reactant produced in hepatocytes, has been used as a severity marker in CAP patients [17]. Arinzon et al. conducted a retrospective study comparing laboratory CRP levels to NHAP severity indexes in predicting short-term mortality. The researchers showed that serum CRP levels taken at the time of a pneumonia diagnosis were better predictors of severity than NHAP prognostic scoring tools [17]. Yet, the study was based on a small participant size and was retrospective in methodology. Larger prospective studies are warranted to fully establish the predictive role of CRP in NHAP severity. Importantly, a 2004 update for guidelines of the British Thoracic Society (BTS) suggested that CRP should not be used as a marker for pneumonia severity since an elevation in CRP is not specific and has no direct correlation to the severity of pneumonia [68–70]. Thus, CRP still remains a questionable biomarker and may have limited utility as a predictor of NHAP mortality.

Copeptin is the C-terminal portion of arginine vasopressin (AVP), a stress hormone originating in the hypothalamus, that is released from the posterior pituitary during periods of stress (i.e., illness or stressor acting on the HPA axis) that has been reported in CAP patients. Copeptin was found to be associated with 30-day mortality in NHAP patients in a prospective study of 73 patients in a Korean hospital by Kim et al. [67] and showed a stronger prediction value than procalcitonin and pro-ANP [71–73]. The authors found that copeptin was on a par with CURB-65 in its predictive capability. Kim et al. [67] attribute the copeptin release in NHAP patients to conditions such as sepsis and heart failure, which characterize these patients [74, 75]. While study has several strengths, including its prospective nature, it has several notable weaknesses: it was conducted in a Korean hospital which may have specific demographic traits limiting its generalizability, reliance solely on chest X-ray for diagnosis (excluding those who did not receive X-ray), influence of DNR orders, and patient refusal of aggressive treatment [67]. This is the only study to date that has examined the role of copeptin in NHAP. More studies are needed with larger sample sizes to fully elucidate the potential of copeptin in the 30-day prediction of NHAP.

### 4.7. Strengths and Limitations

Our study has a notable strength; the study protocol yielded studies that have been conducted all over the world, providing an international flavor of NHAP mortality and its predictors. However, several limitations of our study must be acknowledged. First, we utilized only three search engines that were readily accessible: PubMed (MEDLINE), EMBASE, and CINAHL. Studies reporting issues other than NHAP in the elderly population were excluded from our set. Yet, it may be possible that some studies that investigated other aspects of pneumonia for the elderly unrelated to nursing homes also investigated NHAP and thus were not part of our analysis.

As mentioned, our search yielded several studies from around the world, to provide a global picture of region-specific findings pertaining to NHAP mortality. It is challenging to develop an understanding of NHAP mortality predictors by evaluating research based in different parts of the world, in part due to the variety of microbial etiology. Additionally, our search may not have employed the most optimal keywords to generate an ideal sampling from around the world. A portion of our search included the key words “United States,” “nursing home,” “acquired,” “pneumonia,” and “epidemiology.” Thus, this search through one of the databases may have yielded more US based studies, potentially limiting the generalizability of the outcomes. We conducted a qualitative review and could not perform the meta-analysis given the heterogeneity of the studies included in the analysis.

### 4.8. Directions for Future Research

While prognostic factors of CAP have been studied extensively, the identification of mortality prediction tools for NHAP is an emerging area of research. There is a need to develop adequate clinical prediction tools that take into account patients' functional status. Further studies are also warranted in assessing the CURB score for NHAP patients under age 65. Future studies are needed to determine whether the CURB-65 score is the best approach to a nursing home population that is becoming increasingly younger.

There is also a need to evaluate potential combinations of prognostic tools and biomarkers and their combined predictive value in determining NHAP severity and outcomes. For instance, perhaps the most effective clinic judgment tool entails both a scoring system and a biomarker or even takes into account certain clinical attributes. In one prospective cohort study of 36 patients, Menéndez et al. [52] showed the greatest predictive value when two predictive scales (PSI and CURB65/CRB65) along with C-reactive protein were employed for the 30-day mortality prediction in CAP. Thus, the best possible prognostic indication scheme may entail a new aggregate scoring methodology that, for example, takes into account a subscoring system, one biomarker, a scoring scale, and specific clinical details. Larger, multicenter randomized controlled prospective studies are needed to determine the effectiveness of such a scheme. Additionally, specific subpopulations within the elderly need further investigation to determine differences in risk stratification and mortality predictors.

## 5. Conclusion

NHAP remains an important medical issue in the elderly, particularly due to the rise in the nursing home patient population. There is a need for prognostication guidelines for NHAP as severity prediction tools can aid in the allocation of medical resources and translate to improve clinical outcomes. Clinical assessment tools such as CURB-65, PSI, and SOAR used for predicting severity in CAP have been applied to NHAP. However, they are often intricate in their application and may be marred by inaccuracies due to the frequent lack of presenting symptoms in NHAP patients.

Our study showed that the current evidence points to PSI as having superior predictive value compared to other clinical tools in determining NHAP mortality, with CURB-65 also validated as another very useful tool. The next best tool supported by evidence is SOAR, which may have particular benefit in identifying patients in need of ICU admissions. The evidence in support of biomarkers for predicting NHAP mortality is not completely conclusive, but CRP appears to be the biomarker with the most concrete support. Additionally, copeptin may serve as a reliable predictor of 30-day mortality in NHAP, but more studies are needed to validate its effects and use. No single set of clinical characteristics, prognostic scoring tools, or biomarkers have overwhelming support in their use with NHAP; further prospective studies are warranted in large samples that can delineate the most effective predictor or combination scheme of predictors to ultimately aid clinicians in determining relative likelihood of NHAP mortality.

## Figures and Tables

**Table 1:** Search strategy and keywords employed for the systematic literature search.

Database	Search	Keywords/query
PubMed	1	Nursing home acquired pneumonia risk factors
	2	Nursing home acquired pneumonia predictors
	3	Nursing home acquired pneumonia United States epidemiology

EMBASE	1	Nursing AND home AND acquired AND pneumonia AND risk AND factors
	2	Nursing AND home AND acquired AND pneumonia AND outcome
	3	Nursing AND home AND acquired AND pneumonia AND United AND States AND epidemiology

CINAHL	1	Nursing home acquired pneumonia

**Table 2:** Synopsis of included papers.

Author	Type of study	Patients (*n*)	Risk factors for NHAP	Predictors of NHAP severity
Arinzon et al. [17]	Retrospective study	148	Presence and duration of fever, respiratory rate, serum CRP and albumin levels, lymphocyte count, number of comorbid diseases, CHF, and DM	PSI (Pneumonia Severity Index), Missouri study index, and the nursing home associated pneumonia (NHAP) severity index

Chen [76]	Prospective cohort study	233	Hemoglobin A1c	Barthel index (BI), Charlson comorbidity index (CCI)

Chosa [77]	Prospective study	161	Swallowing impairment	Pneumonia incidences

El Solh et al. [16]	Retrospective study	334	2003 community-acquired pneumonia (CAP) guideline versus the 2005 healthcare-associated pneumonia (HCAP) guideline	Pneumonia severity, multilobar involvement, clinical stability, length of hospital stay, and mortality

Gryglewska et al. [78]	Prospective study	189	Higher diastolic BP, nitrate, and CCB use	Incidence of nursing home-acquired pneumonia among long-term care facilities residents

Gussoni et al. [79]	Prospective study	1,219	Nursing home acquired versus community acquired	Failing therapy

Hutt et al. [80]	Retrospective chart review	2,251	Not using explicit criteria defined by a multispecialty panel	Modified Delphi approach with an episode score that considered hospitalization and timing, route, duration, and spectrum of antibiotics

Hutt et al. [81]	Quasiexperimental, mixed-methods multifaceted intervention trial	1123	Residents not complying with vaccination guidelines	Hospitalization rates

van der Steen et al. [82]	Prospective Study	374	Severity of dementia, swallowing disturbance, aspiration, insufficient food intake, weight loss, and dehydration	Death (rate), cure rate, and increase in discomfort at the onset of pneumonia

Kim et al. [67]	Prospective study	73	Procalcitonin, pro-ANP, and copeptin	Mortality in NHAP

Klapdor et al. [25]	Retrospective study	618	Age	Short- and long-term mortality

Lee et al. [10]	Retrospective observational study	208	N/A	NHAP model score, Pneumonia Severity Index (PSI), CURB-65, and SOAR

Loed [32]	Retrospective study	N/A	Alcoholism, bronchial asthma, immunosuppression, lung disease, heart disease, institutionalization, and increasing age	Prevalence of NHAP

Ma et al. [83]	Prospective study	772	N/A	Sputum routine and mycobacterial cultures, blood and urine cultures, serology, and nasopharyngeal aspirate viral culture and polymerase chain reaction tests

Ma et al. [84]	Prospective, observational cohort study	488	Multidrug-resistant (MDR) bacteria and nursing home-acquired versus community acquired	Katz score, Charlson comorbidity index (CCI), pneumonia severity (CURB score), microbiology, and clinical outcome

Man et al. [85]	Prospective observational study	767	N/A	PSI, CURB-65, M-ATS, R-ATS, and Espana rule

Marrie and Blanchard [28]	Prospective study	243	NHAP versus CAP	Mortality rate

Mylotte et al. [86]	Prospective study	158	N/A	Pneumonia prognosis index

Naughton et al. [54]	Prospective cohort study	378	N/A	Respiratory rate >30 breaths/minute, pulse >125 beats/minute, altered mental status, and a history of dementia

Sund-Levander et al. [87]	Retrospective study	234	Chronic obstructive pulmonary disease, ADL status >5, and male gender	Case-fatality rate

N/A: not applicable.
